# [Retracted] Long non‑coding RNA CRNDE regulates cell proliferation, migration, invasion, epithelial‑mesenchymal transition and apoptosis in oral squamous cell carcinoma

**DOI:** 10.3892/ol.2024.14535

**Published:** 2024-06-27

**Authors:** Jing Dai, Jing-Wen Mu, Hong Mu

Oncol Lett 17: 3330–3340, 2019; DOI: 10.3892/ol.2019.9978

Following the publication of this paper, it was drawn to the Editor's attention by a concerned reader that certain of the immunofluorescence data shown in Fig. 5 on p. 3337 and immunohistochemical data in Fig. 6D on p. 3338 had already appeared in previously published articles written by different authors at different research institutes. Owing to the fact that the contentious data in the above article had already been published prior to its submission to *Oncology Letters*, the Editor has decided that this paper should be retracted from the Journal. The authors were asked for an explanation to account for these concerns, but the Editorial Office did not receive a reply. The Editor apologizes to the readership for any inconvenience caused.

